
JOURNAL INFORMATION
==============================
NLM Title Abbreviation: Age Ageing
Journal ID: Age Ageing
NLM Journal ID: 0375655
Journal ID: ageing

Age and Ageing

ISSN: 0002-0729
EISSN: 1468-2834

ARTICLE INFORMATION
==============================
PMCID: PMC8083189
PMID: 33878163
DOI: 10.1093/ageing/afab070
Article ID: afab070
Article version: 1
Subjects: Editorial, AcademicSubjects/MED00280

Models will only get us so far: planning for place of care and death

Borgstrom Erica The Open University - School of Health, Wellbeing and Social Care Milton Keynes, United Kingdom (ORCID 0000-0002-1009-2928)

Address correspondence to: erica.borgstrom@open.ac.uk
Electronic publication date: 2021 Apr 20
Electronic Location ID: afab070
Copyright: © The Author(s) 2021. Published by Oxford University Press on behalf of the British Geriatrics Society.
Copyright year: 2021
License: This is an Open Access article distributed under the terms of the Creative Commons Attribution License (http://creativecommons.org/licenses/by/4.0/), which permits unrestricted reuse, distribution, and reproduction in any medium, provided the original work is properly cited.
License URL: https://creativecommons.org/licenses/by/4.0/

Keywords: Older people, advance care planning, end-of-life care, Covid-19
Page count: 5
edited-state: accepted-manuscript
article-lifecycle: PAP

==============================
### Models will only get us so far: planning for place of care and death

Author: Dr Erica Borgstrom, The Open University - School of Health, Wellbeing and Social Care

Milton Keynes, United Kingdom (ORCID 0000-0002-1009-2928)

erica.borgstrom@open.ac.uk

Declaration of Conflicts of Interest: None

Declaration of sources of funding: None

Keywords: Older people, advance care planning , end-of-life care , Covid-19

Key points:

- Models for advance care planning can help facilitate decision-making
- For models to be effective, they require people have appropriate skills, knowledge and resources
- Advance care planning has the potential to consider more than just place of care and death

UNCORRECTED MANUSCRIPT

© The Author(s) 2021. Published by Oxford University Press on behalf of the British Geriatrics Society.

UNCORRECTED MANUSCRIPT

Over several decades, there has been increasing attention given to how, as a society and as individuals, we can ensure that people age and die well. On the latter point, end-of-life care policy has focused on place of death as a mechanism for and measure of improving the quality of care [1]. Being cared for and dying in one's usual place of residence, including a care home, has been presumed to be both desirable and beneficial for dying people, those who care for them, and the bereaved [2]. Nevertheless, operationalising this ambition across all groups and geographical areas has been difficult, not least because not everyone has the same preference and/or access to resources [3]. Moreover, the assumptions about the importance of 'dying at home' have been challenged [4].

Amongst this backdrop of thinking about if and how the location of care and death may matter, in countries like the UK, where the death toll from the coronavirus pandemic (COVID-19) has been high and ever present, societally and professionally we have been further challenged to reappraise how care is provided. Considering this, in this issue West and colleagues present a rapid review of reviews to understand place of care and death for older people and how this can be applied to current and future contexts [13]. They ask two main questions: what influences someone's decisions about place and what influences the processes and outcome of place.

Importantly, place of care and place of death can indeed be different places for the same individual as found by West *et al*. There may be multiple places of care, and a change of preferences, across the time someone is at the end of their life. Nevertheless, place of care and place of death are often conflated and viewed as static in the literature and indeed, this can also be seen in patient notes, audit documents, or policy statements. Additionally, whilst home is often the presumed preference, there is considerable heterogeneity and care-givers may not be accurate proxies [5]. Through their review, West *et al*. highlight this variety and the importance of determining if preferences vary.

Consequently, they've developed a decision-making model to support care provision aligned with people's preferences which can be used both during the current pandemic and in other circumstances. Whilst their review was limited to research concerning older people, their model has general relevance when considering end-of-life care.

What underlies much of the literature considered by West *et al*., and indeed the model presented, is a logic of advance care planning and patient choice [6,7]. At its core, this logic presumes that considering and discussing preferences – for example around preferred place of death – can enable patients, informal carers and care professionals to align care provision to the preference [8]. The decision-making model presented here suggests ways in which to support these elements with an emphasis on the information people may need, including information that is accessible, culturally appropriate and in a language the person can understand. The model also encourages considering the decision-making context, such as capacity and the network of people involved. Whilst they do not go as far as to suggest relational autonomy, there is an understanding that some of the decisions being made are not just about the older person. This is evident in their example of how advance care plans may need to be renegotiated and reconsidered as care options change during the COVID-19 pandemic, such as the impact of visitor restrictions. Others have also recommended thinking about joint discussions and plans to acknowledge the COVID-19 context [9,10].

Advance care planning is an evolving field [11] and this may be a useful model for many for capturing a variety of aspects to be considered during the ongoing process of decision-making. However, like most models, it will not in itself do the work. It requires people being willing or able to articulate their preferences – and some may not wish to do this or may consider the future differently to how advance care planning orients time. As seen in other studies about shared decision-making and advance care planning, for it to be effective, those employing this model still require skills and judgment on how to communicate, how to weigh up different factors and manage potential tensions between these elements and/or between people. It takes a commitment to have conversations early, revisit decisions once they have been made and accept that they can be fluid, which has not always been done before [12]. Moreover, for the outcomes to be realised, there will need to be an ongoing effort to ensure the preferences and decisions can be enacted even if they change: assessing needs, providing supports, facilitating adaptation. Patient, families, carers and

UNCORRECTED MANUSCRIPT

professionals therefore not only need access to information, but also a range of skills and resources that facilitate making preferences a reality.

If aligning place of care and death with the dying person's preference is considered an outcome, the process of advance care planning can be a useful mechanism for determining and preparing for such preferences. However, place is not the only 'outcome' that matters to many people – they may be more concerned about how they are cared for, or who they can see, or how much control they feel they have. Advance care planning can consider a much wider remit than just location of care, and it may be the processes of discussion that most beneficial for some. Importantly, decisions – in how they are made, enacted, and experiences – are inherently relational, involving and impacting the dying person and also those who care about them and for them, including health and social care professionals. There is therefore further scope to consider what good outcomes look like for this wider network and how to support them all through the changing contexts of care during the end of life and the COVID-19 pandemic. Models can be useful to support conversations but they will only get us so far.

1. Cox K, Moghaddam N, Almack K *et al.* Is it recorded in the notes? Documentation of end-of-life care and preferred place to die discussions in the final weeks of life. *BMC Palliat Care* 2011;**10**:18.

2. Pollock K. Is home always the best and preferred place of death? *BMJ* 2015;**351**:h4855.

3. Davies JM, Sleeman KE, Leniz J *et al.* Socioeconomic position and use of healthcare in the last year of life: A systematic review and meta-analysis. Prigerson HG (ed.). *PLOS Med* 2019;**16**:e1002782.

4. Robinson J, Gott M, Gardiner C *et al.* The 'problematisation' of palliative care in hospital: an exploratory review of international palliative care policy in five countries. *BMC Palliat Care* 2016;**15**:64.

5. Hoare S, Morris ZS, Kelly MP *et al.* Do Patients Want to Die at Home? A Systematic Review of the UK Literature, Focused on Missing Preferences for Place of Death. Quinn TJ (ed.). *PLoS One* 2015;**10**:e0142723.

6. Borgstrom E. Planning for an (un)certain future: Choice within English end-of-life care. *Curr Sociol* 2015;**63**, DOI: 10.1177/0011392115590084.

7. Mol A. *The Logic of Care: Health and the Problem of Patient Choice*. Abingdon: Routledge, 2008.

8. Thomas K, Lobo B, Detering K. *Advance Care Planning in End of Life Care* . Oxford: Oxford University Press, 2017.

9. Abel J, Kellehear A, Millington Sanders C *et al.* Advance care planning re-imagined: a needed shift for COVID times and beyond. *Palliat Care Soc Pract* 2020;**14**:263235242093449.

10. Hopkins SA, Lovick R, Polak L *et al.* Reassessing advance care planning in the light of covid-19. *BMJ* 2020;**369**:m1927.

11. Rietjens J, Korfage I, Taubert M. Advance care planning: the future. *BMJ Support Palliat Care* 2020:bmjspcare-2020-002304.

12. Pollock K, Seymour J. Reappraising 'the good death' for populations in the age of ageing. *Age Ageing* 2018;**47**:328–30.

UNCORRECTED MANUSCRIPT

13. Emily West, Kirsten Moore, Nuriye Kupeli, Elizabeth L Sampson, Pushpa Nair, Narin Aker, Nathan Davies, Rapid review of decision-making for place of care and death in older people: lessons for COVID-19, *Age and Ageing*, Volume 50, Issue 2, March 2021, Pages 294–306,

UNCORRECTED MANUSCRIPT
